# Rheumatism

**Published:** 1869-08

**Authors:** 


### Rheumatism.

At St. Mary's Hospital the treatment, as adopted by Dr. Gibson, may be described as embracing three points: 1st, removal of pressure and tension of joints; 2d, an even and warm temperature; 3d, removal or relief of pain. To accomplish the first of these ends, the patient lies in bed, and his joints muffled in cotton, wool and flannel, a cradle being placed where the weight of the bed-clothes is painful. For the second, the patient wears a flannel dressing-gown, and the blankets touch the skin of the lower extremities, sheets being placed over only the upper part of the bed. For the third, the linimentum belladonnæ (ext. belladon. ʒ j., linim. saponis ℥ vj.) is applied to painful joints, and covered over with wadding. Occasionally, where the pain is very excessive, from an eighth to a quarter of a grain is injected subcutaneously. For the rest, he has now and then found it useful to apply a leech or two to a swollen joint, or to the cardiac region. In cases where there appears to be a gouty complication, Dr. Gibson employs a little iodide of potassium; but apart from this he does not give any potash to his patients. He rarely finds the urine containing acid after the first few days of treatment. As regards food, the patient is allowed from the first roast meat, rice-pudding and porter. The patients spoke in strong terms of the efficacy of the belladonna liniment. At night they generally slept well.—*Lancet.*

